# Systemic Inertia in Addiction Care: Institutional Hesitancy and Ethical Barriers to Buprenorphine Initiation

**DOI:** 10.7759/cureus.96971

**Published:** 2025-11-16

**Authors:** Edicer Ramírez-Rivera, Sofía Bravo-Torres, Alana N Ortiz-Fraguada, Karen G Martínez-González

**Affiliations:** 1 Department of Psychiatry, University of Puerto Rico, Medical Sciences Campus, San Juan, PRI

**Keywords:** buprenorphine initiation, ethics, gatekeeping, institutional anxiety, patienthood

## Abstract

This editorial examines a case illustrating the ethical tensions that arise when systemic uncertainty and institutional anxiety delay evidence-based treatment for opioid use disorder (OUD). A patient admitted through an emergency department received buprenorphine only after a delay between order and administration, reflecting the institution’s limited readiness for medication for OUD (MOUD) implementation. Although protocols existed, they were inconsistently applied. The commentary explores how surveillance-oriented systems, stigma, and fear of regulatory scrutiny transform care into cautious oversight. It argues that moral readiness for MOUD requires institutional trust, interprofessional collaboration, and procedural clarity to ensure that fear of error never outweighs the ethical imperative to relieve suffering and save lives.

## Editorial

A 42-year-old man arrived at the emergency department (ED) in opioid withdrawal, agitated, and in pain from a chronic, malodorous ulcer on his left leg related to injection use. His history was notable for opioid use disorder (OUD), a chronic and treatable medical condition involving compulsive substance use despite harmful consequences [1]. Psychiatry was consulted by the ED team to evaluate the patient under emergency provisions of Puerto Rico’s mental health law and to guide withdrawal management.

At presentation, the patient had received haloperidol 10 mg IM, lorazepam 2 mg IM, and diphenhydramine 50 mg IM for agitation and acute psychosis. Internal medicine initiated medical stabilization with intravenous fluids, wound care, and empiric broad-spectrum antibiotics, including vancomycin 1.25 g IV every 12 hours, pending infectious disease recommendations. Laboratory and imaging evaluations included renal and abdominal ultrasounds to assess for potential end-organ or infectious complications associated with chronic opioid use and repeated injections. Additionally, left-leg imaging was obtained to rule out osteomyelitis. The patient was monitored for opioid withdrawal and other complications while coordination between the ED and consulting medical teams continued.

Once medically stable and more alert, the patient described worsening withdrawal symptoms with a Clinical Opiate Withdrawal Scale (COWS) score greater than eight and agreed to initiate treatment for opioid dependence. He was then admitted under the internal medicine service for continued management, with psychiatry providing daily consultation for withdrawal treatment and behavioral stabilization. Psychiatry recommended and ordered buprenorphine, an evidence-based medication for opioid use disorder (MOUD), starting with 4 mg sublingual for induction and an additional 4 mg as needed based on withdrawal severity, with ongoing reassessment and vital sign monitoring every two hours to guide subsequent dosing adjustments.

However, administration was significantly delayed as clarification was sought about ordering pathways, oversight, and documentation. Despite the order being placed promptly, the patient went nearly a full day without receiving either the initial 4-mg induction dose or the scheduled follow-up dose. During this delay, his withdrawal symptoms worsened, with a COWS score greater than 10.

At one point, a staff member asked, “Does the patient really need that medication?”, a question that captured the institution’s broader discomfort with providing addiction treatment as part of routine inpatient care. Buprenorphine was subsequently reordered at 8 mg sublingual daily and finally administered. Although a protocol for buprenorphine initiation existed, it was inconsistently applied, and staff familiarity varied. The medication had been ordered by the psychiatry team, but the delay reflected institutional uncertainty and workflow gaps, including pharmacy hesitation, nursing inaction when the order was not executed, and lack of follow-up by the primary service, rather than disagreement among clinical teams.

When buprenorphine was eventually administered, the patient’s symptoms improved rapidly. The case underscored a gap between clinical intention and institutional readiness. While the patient ultimately benefited, the delay illustrated how procedural caution can inadvertently perpetuate suffering, especially when addiction treatment remains underutilized in hospital settings. Federal guidelines emphasize the importance of initiating medications for OUD during hospitalization and ensuring continuity of care after discharge [2-5].

Patienthood and conditional care

“Patienthood” refers to the moral and social recognition that grants individuals legitimacy as recipients of care [6]. For people with OUD, that legitimacy is often conditional. Stigma and societal narratives of self-blame can cause institutions to approach addiction treatment as discretionary rather than standard medical care [7,8].

Buprenorphine is a partial μ-opioid receptor agonist and κ-antagonist that alleviates withdrawal and craving with a lower risk of respiratory depression. Methadone, in contrast, is a full μ-opioid receptor agonist that requires administration in structured opioid treatment programs [9,10]. Ethnographic and qualitative evidence shows that patients who use buprenorphine or methadone frequently experience discrimination or mistrust in clinical settings, which compromises engagement and continuity [11,12].

Although buprenorphine offers a safer profile and can be prescribed in general medical settings, its use often meets hesitation due to stigma, concerns about diversion, and perceived regulatory complexity. When clinicians or systems hesitate to implement MOUD despite clear evidence of benefit, they signal uncertainty about whether these patients are “deserving” of the same timely care provided for other chronic illnesses.

Research on patients’ experiences with diversion and nonprescribed buprenorphine use further reveals that mistrust and structural stigma, rather than concerns about pharmacologic risk, often drive restrictive practices [13]. These forms of moral gatekeeping challenge beneficence and justice by transforming addiction care into a test of trustworthiness rather than an obligation of care.

Health surveillance and systemic anxiety

Health surveillance frameworks, meant to safeguard safety and accountability, can foster systemic anxiety that delays ethical action [14,15]. The question, “Does the patient really need that medication?” illustrates how institutional worry can transform an evidence-based prescription into a moral query. The delay in this case stemmed from an institution-wide unease about compliance, oversight, and workflow clarity. Barriers to MOUD implementation frequently include regulatory fear, limited institutional support, and uncertainty about professional roles [16]. Ongoing clinician discomfort and confusion regarding prescribing requirements persist even after federal training rules were eased [17].

Under current federal policy, any clinician with a valid Drug Enforcement Administration (DEA) registration may initiate buprenorphine for OUD without a waiver, in both inpatient and outpatient settings. Federal guidance, including the Substance Abuse and Mental Health Services Administration’s *Federal Guidelines for Opioid Treatment Programs* [4] and workforce recommendations by the U.S. Department of Health and Human Services [5], emphasizes standardized protocols, documentation, and linkage to community care. Institutional anxiety often arises from perceived regulatory scrutiny, pharmacy audits, and workforce shortages despite these allowances [2,3].

In Puerto Rico, MOUD delivery remains uneven. Urban areas, such as the San Juan metropolitan region, have greater service availability; however, implementation remains inconsistent, and continuity after discharge is limited. Rural regions face additional barriers related to stigma, transportation, and limited treatment infrastructure [18]. Nationally, only about 17 percent of individuals with OUD received medication treatment in 2024 [19]. The proportion in Puerto Rico is likely lower. This gap highlights the urgent need for health institutions on the island to operationalize evidence-based care.

Institutional vigilance can resemble the system-level barriers that hinder addiction treatment in hospitals, such as ambiguous leadership, unclear workflows, and siloed communication [20]. Regulatory complexity within opioid treatment program oversight can further heighten institutional fear, leading hospitals to delay or avoid buprenorphine initiation despite clear evidence of its safety and necessity [4]. When fear of oversight outweighs the duty to relieve suffering, health systems risk replicating the distrust that marginalized patients already encounter outside the hospital.

Iatrogenic anxiety and ethical harm

Worry itself can become a byproduct of modern health systems, particularly when institutional caution transforms routine care into moral uncertainty. In addiction treatment, anxiety is reciprocal: patients fear abandonment, clinicians fear reprimand, and administrators fear regulatory scrutiny [21]. These intersecting worries generate what Buchbinder et al. term *moral stress*, a state that arises when organizational barriers prevent clinicians from acting in accordance with their ethical duties [22].

These dynamics have measurable consequences for both patients and institutions. Evidence suggests that early initiation of buprenorphine reduces emergency visits and mortality [23,24]. In U.S. cohorts, buprenorphine pharmacotherapy has been linked to more than a fourfold lower risk of suicide or overdose death [25] and a 62 percent reduction in opioid-involved mortality following a nonfatal overdose [26]. Modeling studies further estimate that buprenorphine reduces opioid overdose deaths by approximately 18% over ten years compared with no treatment, with evidence that higher daily doses offer additional protection [27,28].

Yet fear-driven hesitation undermines beneficence. Recent reviews and clinical guidelines affirm that hospitals should view medications for OUD as routine, life-saving care rather than as exceptional interventions [2,3]. Calcaterra et al. likewise report that failure to initiate buprenorphine before discharge correlates with higher relapse and overdose risk [29].

In practice, what begins as procedural hesitation can erode compassion and moral clarity. This case exemplifies harm through omission: procedural delay became a form of iatrogenic anxiety that affected both patient and staff. When systems hesitate, patients internalize institutional doubt as self-doubt, reinforcing stigma and weakening trust.

Toward ethical and moral readiness

Moral readiness for MOUD implementation requires alignment between clinical judgment, organizational support, and professional education. Workforce shortages, reimbursement uncertainty, and training limitations persist as significant obstacles [30]. Even after regulatory changes, many waivered clinicians prescribe infrequently, reflecting ongoing institutional barriers [31]. Addressing these challenges demands cultural transformation, not just policy change.

Achieving moral readiness depends on translating institutional commitment into coordinated practice. Clinicians consistently emphasize the value of interprofessional collaboration and clear linkage between inpatient and outpatient care, yet system-level inertia often prevents consistent follow-through [32]. Pharmacists play a critical but underutilized role in sustaining continuity of care and reducing prescribing hesitancy [33]. Hospital-wide initiatives, such as standardized protocols, staff education, and addiction-consult services, have been shown to substantially increase buprenorphine initiation rates [34]. Readiness, therefore, is not merely procedural but ethically grounded in shared accountability and the collective obligation to act.

Professional consensus holds that hospital-based teams bear an ethical responsibility to treat substance use disorder as core medical care rather than as an ancillary service [35]. Institutions must shift from defensive oversight to collaborative trust. Federal guidance now explicitly calls for the availability of MOUD across inpatient and outpatient settings, recognizing untreated withdrawal as a violation of both ethical and clinical standards [36]. Ethical readiness, therefore, means acting decisively despite uncertainty, treating institutional anxiety as a signal for reflection rather than paralysis.

Reframing readiness

This case illustrates how systemic anxiety and procedural ambiguity distort ethical priorities in OUD care. The delay in buprenorphine administration stemmed not from clinical disagreement but from institutional unpreparedness to treat addiction as routine medicine. When systems prioritize risk management over compassion, patienthood becomes conditional, and trust erodes. Recognizing institutional worry as an opportunity for moral growth allows health systems to reframe surveillance as stewardship. Timely MOUD initiation is not optional; it is an ethical obligation.

## References

[1] Taylor JL, Samet JH (2022). Opioid use disorder. Ann Intern Med.

[2] Funk MC, Nash S, Smith A (2023). Treatment of opioid use disorder in the general hospital. Am J Psychiatry.

[3] Englander H, Thakrar AP, Bagley SM, Rolley T, Dong K, Hyshka E (2024). Caring for hospitalized adults with opioid use disorder in the era of fentanyl: a review. JAMA Intern Med.

[4] (2024). Federal Guidelines for Opioid Treatment Programs. Rockville, MD: SAMHSA.

[5] (2023). Overdose Prevention Strategy. Washington, DC: U.S. Department of Health and Human Services.

[6] Parsons T (1951). The Social System. Glencoe, I.L. (ed): Free Press.

[7] Corrigan PW, Rao D (2012). On the self-stigma of mental illness: stages, disclosure, and strategies for change. Can J Psychiatry.

[8] Livingston JD, Milne T, Fang ML, Amari E (2012). The effectiveness of interventions for reducing stigma related to substance use disorders: a systematic review. Addiction.

[9] Schuckit MA (2016). Treatment of opioid-use disorders. N Engl J Med.

[10] Falcon E, Browne CA, Leon RM, Fleites VC, Sweeney R, Kirby LG, Lucki I (2016). Antidepressant-like effects of buprenorphine are mediated by kappa opioid receptors. Neuropsychopharmacology.

[11] van Boekel LC, Brouwers EP, van Weeghel J, Garretsen HF (2016). Experienced and anticipated discrimination reported by individuals in treatment for substance use disorders within the Netherlands. Health Soc Care Community.

[12] Abadie R, McLean K, Habecker P, Dombrowski K (2021). Treatment trajectories and barriers in opioid agonist therapy for people who inject drugs in rural Puerto Rico. J Subst Abuse Treat.

[13] Sud A, Salamanca-Buentello F, Buchman DZ, Sabioni P, Majid U (2022). Beyond harm-producing versus harm-reducing: a qualitative meta-synthesis of people who use drugs' perspectives of and experiences with the extramedical use and diversion of buprenorphine. J Subst Abuse Treat.

[14] Armstrong D (1993). Public health spaces and the fabrication of identity. Sociology.

[15] Rose N (2001). The politics of life itself. Theory Cult Soc.

[16] Mackey K, Veazie S, Anderson J, Bourne D, Peterson K (2020). Barriers and facilitators to the use of medications for opioid use disorder: a rapid review. J Gen Intern Med.

[17] Lanham HJ, Papac J, Olmos DI, Heydemann EL, Simonetti N, Schmidt S, Potter JS (2022). Survey of barriers and facilitators to prescribing buprenorphine and clinician perceptions on the Drug Addiction Treatment Act of 2000 waiver. JAMA Netw Open.

[18] (2025). Substance Abuse and Mental Health Services Administration. Key substance use and mental health indicators in the United States: Results from the 2022 National Survey on Drug Use and Health. HHS Publication No. PEP23-07-01-006, NSDUH Series H-58. Center for Behavioral Health Statistics and Quality, Substance Abuse and Mental Health Services Administration. https://www.samhsa.gov/data/sites/default/files/reports/rpt42731/2022-nsduh-nnr.pdf.

[19] (2025). Substance Abuse and Mental Health Services Administration. Key substance use and mental health indicators in the United States: Results from the 2024 National Survey on Drug Use and Health. HHS Publication No. PEP25-07-007, NSDUH Series H-60. Center for Behavioral Health Statistics and Quality, Substance Abuse and Mental Health Services Administration. https://www.samhsa.gov/data/sites/default/files/reports/rpt56287/2024-nsduh-annual-national-report.pdf.

[20] Englander H, Jones A, Krawczyk N, Patten A, Roberts T, Korthuis PT, McNeely J (2022). A taxonomy of hospital-based addiction care models: a scoping review and key informant interviews. J Gen Intern Med.

[21] Hoffman KA, Ponce Terashima J, McCarty D (2019). Opioid use disorder and treatment: challenges and opportunities. BMC Health Serv Res.

[22] Buchbinder M, Browne A, Berlinger N, Jenkins T, Buchbinder L (2024). Moral stress and moral distress: confronting challenges in healthcare systems under pressure. Am J Bioeth.

[23] D'Onofrio G, O'Connor PG, Pantalon MV (2015). Emergency department-initiated buprenorphine/naloxone treatment for opioid dependence: a randomized clinical trial. JAMA.

[24] Sordo L, Barrio G, Bravo MJ (2017). Mortality risk during and after opioid substitution treatment: systematic review and meta-analysis of cohort studies. BMJ.

[25] Vakkalanka P, Lund BC, Arndt S, Field W, Charlton M, Ward MM, Carnahan RM (2021). Association between buprenorphine for opioid use disorder and mortality risk. Am J Prev Med.

[26] Samples H, Nowels MA, Williams AR, Olfson M, Crystal S (2023). Buprenorphine after nonfatal opioid overdose: reduced mortality risk in medicare disability beneficiaries. Am J Prev Med.

[27] Stringfellow EJ, Lim TY, DiGennaro C (2023). Long-term effects of increasing buprenorphine treatment seeking, duration, and capacity on opioid overdose fatalities: a model-based analysis. J Addict Med.

[28] Lei F, Lofwall MR, McAninch J (2024). Higher first 30-day dose of buprenorphine for opioid use disorder treatment is associated with decreased mortality. J Addict Med.

[29] Calcaterra SL, Lockhart S, Natvig C, Mikulich S (2023). Barriers to initiate buprenorphine and methadone for opioid use disorder treatment with postdischarge treatment linkage. J Hosp Med.

[30] Haffajee RL, Bohnert AS, Lagisetty PA (2018). Policy pathways to address provider workforce barriers to buprenorphine treatment. Am J Prev Med.

[31] Jones CM, Olsen Y, Ali MM (2023). Characteristics and prescribing patterns of clinicians waivered to prescribe buprenorphine before and after release of new practice guidelines. JAMA Health Forum.

[32] Shearer R, Englander H, Hagedorn H (2025). Hospital providers’ perspectives on MOUD initiation and continuation after inpatient discharge. J Gen Intern Med.

[33] Kosobuski L, O'Donnell C, Koh-Knox Sharp CP, Chen N, Palombi L (2022). The role of the pharmacist in combating the opioid crisis: an update. Subst Abuse Rehabil.

[34] Calcaterra SL, Lockhart S, Natvig C, Mikulich-Gilbertson SK (2025). Measuring the impact of a multi-site in-hospital intervention for opioid use disorder treatment provision: a survey of hospital-based clinicians. J Gen Intern Med.

[35] Englander H, Priest KC, Snyder H, Martin M, Calcaterra S, Gregg J (2020). A call to action: hospitalists’ role in addressing substance use disorder. J Hosp Med.

[36] U.S. Department of Justice, Bureau of Justice Assistance; National Institute of Corrections (2023). Guidelines for Managing Substance Withdrawal in Jails: A Tool for Local Government Officials, Jail Administrators, Correctional Officers, and Health Care Professionals. Washington, DC: U.S. Department of Justice, Office of Justice Programs.

